# Research on cognitive evaluation of forest color based on visual behavior experiments and landscape preference

**DOI:** 10.1371/journal.pone.0276677

**Published:** 2022-11-03

**Authors:** Wenyue Lin, Yanxia Mu, Zhe Zhang, Jin Wang, Xiuli Diao, Zijing Lu, Wencheng Guo, Yu Wang, Bo Xu

**Affiliations:** College of Landscape Architecture and Horticulture, Southwest Forestry University, Kunming, China; Chinese Academy of Sciences, CHINA

## Abstract

Forest colors are important elements for public enjoying the scenery. So increasing attention has been acquired on forest color cognition. However researches on relationship between forest colors and public response are still insufficient, which cannot provide sufficient theoretical basis for the regulation of forest landscape in color. Therefore, We seek to examine the relationship between forest color and visual behavior based on eye tracking technology, and further interpret the visual indicators through the value of scenic beauty. This study researched Jiaozi Mountain in China by selecting 29 sampling points, counting up 116 photographs in 4 seasons by a mountainous region. On this basis, Matlab was performed to quantitatively extract color elements, while ArcGIS and Fragstats were applied to extract the spatial index of color patches. A total of 10 indicators were obtained to explain the color characteristics of each forest image. Through both visual behavior experiment and landscape preference evaluation, the results showed that people tend to have different visual behaviors and preference cognition when observing forest colors of different seasonal types. Based on the study of forest landscape color in all seasons, the subjects tend to judge the image in comparison to other seasonal forest landscape color photos to identify it more easily. For a single-season forest colors, diversified color information and abundant visual attention are important factors influencing the correlation between visual behavior, landscape preference, and forest color characteristics. This study aims to further reveal people’s perceptions and psychological preference to forest colors, contribute to the establishment of a more quantitative and scientific scenery evaluation system, and provide a scientific basis for forest color planning and design.

## Introduction

In recent years, the public’s view of forests has gradually expanded from traditional resource utilization to aesthetic, emotional, and psychological satisfaction [1]. Forest recreation has become normal behavior for the Chinese public, especially urban residents. The public’s experience of forest landscapes largely depends on the visual sense. Color perception is the first element of vision, which directly affects the public’s cognitive evaluation of a landscape [2]. Therefore, forest color evaluation has become a research hotspot in the field of landscape visual quality evaluation at home and abroad. In the past three years, the investigation of forest color cognition and preference has attracted more and more attention. At present, the majority of forest color evaluation uses questionnaires, interviews, and other subjective methods; there is a lack of monitoring and analysis of objective physiological indicators [3, 4], and results may be influenced by the different choices of respondents. The spatial scale of the research is of small-and-medium size with as such plant organs, single plant and forest landscape [5]. Whereas, few researches can be found on the ornamental landscape outside the forest and the larger scale forest color [6]. However, landscape colors, such as the surrounding mountains and urban mountain forests, are of great value to the overall urban characteristic environment shaping, which are worthy of attention. There are many studies on forest color in a single season, but it cannot ensure the sustainability of forest color landscape throughout the year. Therefore, the investigation period should be extended to study forest color, and the results can be obtained from the annual color evaluation. The exploration and optimization of the changes in four season color is an important step toward improving the visual quality of forests all year round.

Eye tracking technology provides a new perspective for forest color evaluation [7]. Its evaluation index is based on the human gaze, which is objective and can alleviate the drawbacks of inaccurate subjective evaluation [8]. Visual behavior patterns are closely related to human attention and cognitive processes [9]. Some studies have shown that, when observing landscape images, people’s sight is not random; they often produce their own unique visual navigation mode [10]. Observers adjust their visual behaviors unconsciously to meet the needs of landscape cognition [11]. Therefore, eye tracking technology can help objectively grasp the public’s true reflection of forest landscape, and the results are more reliable [12]. In recent years, more and more attention has been paid to the interaction between human visual behavior and landscapes. Experimenting with only visual orientation is one way Dupont selected people with two different knowledge backgrounds (landscape specialty and non-landscape specialty) as subjects to analyze the difference between the two groups in terms of viewing landscape photos [13]. Liang explored in differences of observers’ attention when watching different landscapes through an eye movement hotspot map. He found that in a natural landscape with high landscape complexity, the subjects’ attention range was wide, and most areas of the whole picture had been observed [14]. From these studies, it can be found that researchers pay more attention to the observer’s gaze behavior data for clearly understanding visual information observers pay more attention to. However, visual indicators can not further indicate the cognition and preference of the subjects. For example, the observer’s gaze on the area for a long time may not necessarily represent his liking, but may also be influenced by irritability or other emotions. Therefore, visual experiments alone often cannot interpret complex landscape problems. So most studies are multiple interaction evaluations. By linking visual experiments with preference, perception and perception experiments, the method forms interactive results, which are mutually validation and interpretation. Scott explored the effectiveness of the visual behavior index in measuring the aesthetic response to landscape photos, and found the visual behavior index was positively correlated with the landscape preference of the participants. The more beautiful the landscape was, the more fixation time was noticed from the observers [15]. In the interactive evaluation of the visual behavior experiment and physiological monitoring, some scholars have studied the influence of seasonal plants color changes in the design of healing gardens for psychiatric patients [3]. There are also some studies on the interactive evaluation of psychological perception under audio-visual interaction. The results showed that a close match between landscape elements and sound sources could effectively alleviate stress for observers [16]. In conclusion, Based on the objective results generated by visual behavior, combined with preference, cognition, physiological and psychological response, it can help to interpret the landscape exactly, which is also the entry point of this paper in landscape evaluation.

Eye tracking technology has been recognized as a mature technology by experts in the field of landscape research [17]. Some scholars have also conducted in-depth exploration on forest landscape. Gao found that observers’ visual behavior was related to the richness of landscape content. Scenes with diverse plant species, rich colors and clear landscape structure were more interesting to observers, and visual behavior tends to focus on local areas [1]. Based on visual behavior indicators, Weng explored the effects of forest-light and backlight on the anxiety and attention of viewers in the same slope direction. The study found that backlit landscapes were better at restoring directed attention and more effective at suppressing negative emotions under induced stress [18]. Some scholars introduced color quantitative indicators to explore the impact of color attributes on visual fatigue. The study found that green color could effectively improve public visual fatigue, and the increase of landscape color difference was more likely to form visual fatigue [19]. Although many scholars have discussed the relationship between plant color changes caused by attribute changes and external factors and human responses, few scholars have explored the effects of plant color attributes on human psychology and physiology from the perspective of homogenous landscape.

It is the key to better understand the relationship between the characteristics and differences of forest colors in different seasons to evaluate human visual behavior and psychological cognition. This is also the first problem to be solved in the practical construction and enhancement of the visual quality of forests throughout the year. In China, some scenic spots, such as Jiaozi Mountain, are favored by tourists for their colorful and beautiful autumn scenery. The attractions of a single season can also be a hot spot for forest tourism. Chinese people pay more and more attention to the improvement of forest landscape around cities. As the main element of forest landscape, the color collocation of plants is very important to improve the quality of forest visual aesthetic. At present, color research has been applied to tree species regulation by some scholars. Chen applied the method of color frequency representation and color composition classification in the architectural color classification method to the color composition analysis of plant communities and optimized them. It was found that when the ratio of main chromatography, selective chromatography and dotted chromatography was 6:3:1, the beauty of plant communities was higher [20]. Mu screened slope forests which were most often designated as superior visual beauty in forest landscape photos of Jiaozi Mountain, and finally selected 9 forests completely covered by typical tree species in the region. The threshold range and combination relationship of color landscape are obtained from the typical dominant forest, so as to realize the control of these color indicators [21]. Thus, the visual evaluation results of forest color are transformed into tree species selection and color fusion. At the same time, in order to achieve the purpose of improving the visual quality of the forest, in the actual process of forest management and forest ecological restoration, the method of forest renewal is often adopted, which usually uses new species to replace the fragile trees. In fact, this can be an effective way to continuously improve the color of urban forests. Therefore, there are the following approaches to further improve the urban forest landscape based on the study of forest color: 1) In the process of barren mountains or wasteland construction, and mountain ecological restoration, the color pattern favored by the public is appropriately used to configure the composition and spatial relationship of tree species. 2) In forest management, seasonal color-changing tree species could be introduced appropriately in thinning or replanting. 3) The above tree species configuration design, or tree replanting strategy, can be prioritized the improvement of forest color in one season, which is the easiest to achieve. In addition, from the perspective of the urban area, when forests in different locations have good landscape in different seasons, the urban forest landscape can also have good visual effects. 4) If there is a wider range of tree species to be replaced, or if there is a greater possibility of tree species being selected for afforestation on barren hills, then the use of more seasonal color species can be achieved. We can combine the different tree species that change color in spring, summer, and autumn in a way the public likes, so that the forests could have good color recognition for two or even three seasons.

From these studies, we can find that scholars mainly evaluate the visual quality of forest landscape in terms of landscape types, landscape attributes, and external factors affecting forest color. Some scholars implemented the forest visual quality to the tree species level through the architectural color classification method and aesthetic evaluation method, and put forward operational tree species selection, replacement and supplement strategies to optimize forest color. For the evaluation method of landscape visual quality, most scholars still adopt subjective evaluation method. Although landscape preference and eye movement method are involved in the construction of fitting models, there are still relatively few analyses on the application of both in the evaluation of homogeneous landscape development and the comparison of evaluation results. The existing studies mainly focus on autumn seasonal landscape color, while the remaining three seasonal color studies are relatively scarce. At present, there is no effective research method to dynamically evaluate the color change process throughout the year. Therefore, this study selected Jiaozi Mountain in Yunnan Province as a sample site and took the whole year as the time scale to clarify the characteristics and differences of forest color landscape in different seasons in human visual behavior and landscape preference evaluation. That is, based on color features, the effects of participants’ cognitive evaluation of forest color on their visual observation behavior were analyzed. And find out the color feature elements that affect the visual behavior, put these color features into the tree species level, and provide the visual aesthetic basis for the forest landscape color improvement of similar plant species. This study is aimed to solve the following issues. 1) The difference of forest color characteristics in Jiaozi Mountain during different seasons. 2) The evaluation and differences of forest color visual behavior and landscape preference in different seasons. That is to say, whether SBE can be used to read visual behavioral indicators. 3) The influence of different class forest color characteristics on visual behavior.

## Materials and methods

### Study area

**Fig 1:**
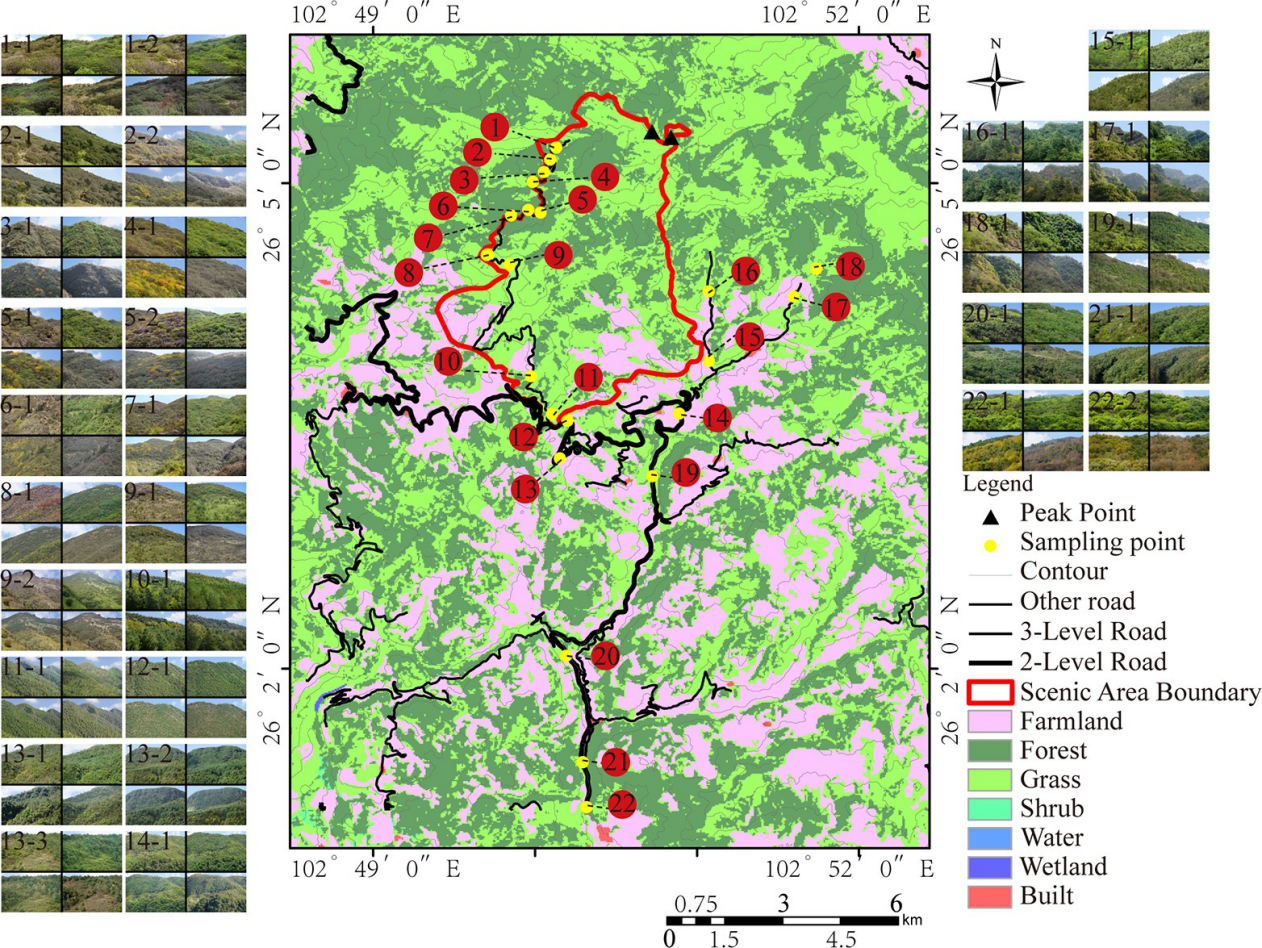
Distribution of sampling points. The land use of Jiaozi Mountain in 2020, with a spatial resolution of 30 x 30, can be divided into seven types: farmland, forest, grassland, shrubland, wetland, water body and built-up area, according to Global 30 (http://www.globallandcover.com/). The distribution vector data of roads in Jiaozi Mountain in 2015 came from the National Catalogue Service for Geographic Information (https://www.webmap.cn.

Jiaozi Mountain Nature Reserve is located in Kunming City, Yunnan Province, China. It is one of the medium-sized nature reserves in China. Because of its unique geographical location, landform, and abundant flora and fauna, it has important scientific research and conservation value in biodiversity conservation and ecological construction in southwest China. The Jiaozi Mountain Reserve, with an altitude of 2300 to 4344 m, has a natural vegetation coverage rate of over 70%. It is typical of the plateau vegetation in central Yunnan, having preserved the most complete vegetation and habitat vertical zone spectrum as well as the most abundant vegetation types. The dominant community types include *Abies georgei var*. *Smithii*, *Sabina squamata*, *Rhododendron simsii*, *Quercus pannosa*, and other plant communities. These plants show different seasonal and phenological characteristics with seasonal changes, forming a rich and colorful four-season forest landscape. Through a comprehensive survey of the Jiaozi Mountain Nature Reserve, we gained a basic understanding of its stand status, tree species types and visual field conditions, and then performed line transect system sampling to select a number of sampling points along the main tour routes of Jiaozi Mountain Nature Reserve. When selecting sample sites along the road, a few hillside plants grow well and there were no factors affecting forest evaluation, such as too much artificial landscape or bare rock soil, etc. In principle, these sites could be selected for our study sample sites. However, due to winding roads and complex terrain in mountainous areas, the observation angle and location were limited to some extent. Influenced by tall plants around roads, we could not obtain complete forest information by taking photos. Therefore, such sites are not included in the study samples. In addition, some slopes of mountains in different slope directions, which were too closed to each other, and the plant composition and color characteristics were too similar, so we made certain screening during field research. Finally, there were a total of 29 observation points with typical forest color characteristics in central Yunnan taken as the sampling points (Fig 1).

### Image acquisition and processing

In previous studies, most scholars have assessed the reliability and sensitivity of landscape photos and slides. Studies have shown that photos and slides have the advantages of easy operation and strong controllability in experiments, and can objectively convey the visual characteristics of a landscape. It has been proven that there is no significant difference between the results of a photo evaluation and a field evaluation [22, 23]. Therefore, in this study, forest color photos were used as experimental materials for an eye movement experiment and landscape preference evaluation.

The time dimension of this study includes four seasons: spring, summer, autumn and winter, with variation in color duration between different sampling points. According to the phenological period and color changes of various tree species in Jiaozi Mountain, the same imaging technology and weather conditions were used to shoot the target forest in the 29 sample sites for multiple times from 9:00 to 16:00 in May 2019 to July 2020 [24]. We took at least one photo from the same angle in each of the 29 sites for 4 seasons. We took photos of the color landscape in spring at the targeted sites from March 19 to March 22, April 16 to 19, and May 1 to 4, 2020. The summer forest color landscape photos were taken from May 31 to June 2, 2019, June 19 to 23, and July 21 to 25, 2020. From September 19 to 22, 2019, and from October 15 to 19, 2019, photos of autumn forest color landscape were taken. Photos of the landscape in winter were taken from December 15 to 19, 2019, January 17 to 21, and February 11 to 16, 2020. In order to avoid the multiple impacts of environmental elements (illumination, color temperature, etc.) and non-stand elements (exposed earth and rock, artificial structures, etc.) on the visual quality of color photos, the comparability of color photos was ensured, while the shooting and processing of photos were standardized. In order to ensure the same visual view of the target forest in the same place for each shooting, GPS (Garmin etrex201x, Shanghai) was applied to record the longitude, latitude and altitude of the sample site, while rangefinder (SNDWAY SW-1000A, Dongguan) was used to measure the relative height, observation distance and perspective of the observer and the target forest. Also, a compass instrument was employed to measure the slope direction of the target forest, and a digital illuminometer (TES-1332A, Taiwan) was used to measure the illumination intensity of the corresponding period. A 24.3-megapixel Sony A6000 camera was relied on to photograph and sample the site at the same position, same direction, same angle and same observation distance at all times. The Jiaozi Mountain Nature Reserve is a complete mountain range with different slope directions. Additionally, sunlight exposure varies between different seasons and different time periods. When the sunlight is in the positive direction, the forest color is shown to be more real. Conversely, the forest color lacks clarity when the Angle of sunlight is in the opposite direction. Therefore, the slope direction, season and sunshine rule will be considered to shoot continuously for 3 to 5 days in practice, so as to shoot the color landscape pictures of the direct sunlight on the target forest.

After the completion of the photo collection, screening and preprocessing were undertaken. The representative photos of forest color with seasonal characteristics were retained, and the photos with uncontrollable factors such as human disturbance and weather conditions were excluded [25]. In order to ensure that we could get the most color representation, we selected the photos with the most flowering in the spring or the most discoloration in the fall from multiple photos taken in each season, as the representative image of the spot in that season. In the end, from 29 sample points, 4 images in spring, summer, autumn and winter were selected to form 116 photos.

### Ethics statement

The Ethics Review Committee of Southwest Forestry University has made an exemption for this experiment. According to relevant Chinese legislation (the Medical Research Involving Human Subjects Act), this visual behavior experiment is a kind of study about subjects’ attitude and viewpoint. Also this experiment did not involve the invasion of personal privacy or the physiological and psychological effects. Before doing this experiment, the subjects had been informed of the relevant experimental procedures and experimental information, and had signed the informed consent for the experimental study on forest color cognition.

### Visual behavior experiments

#### Experimental materials

**Fig 2:**
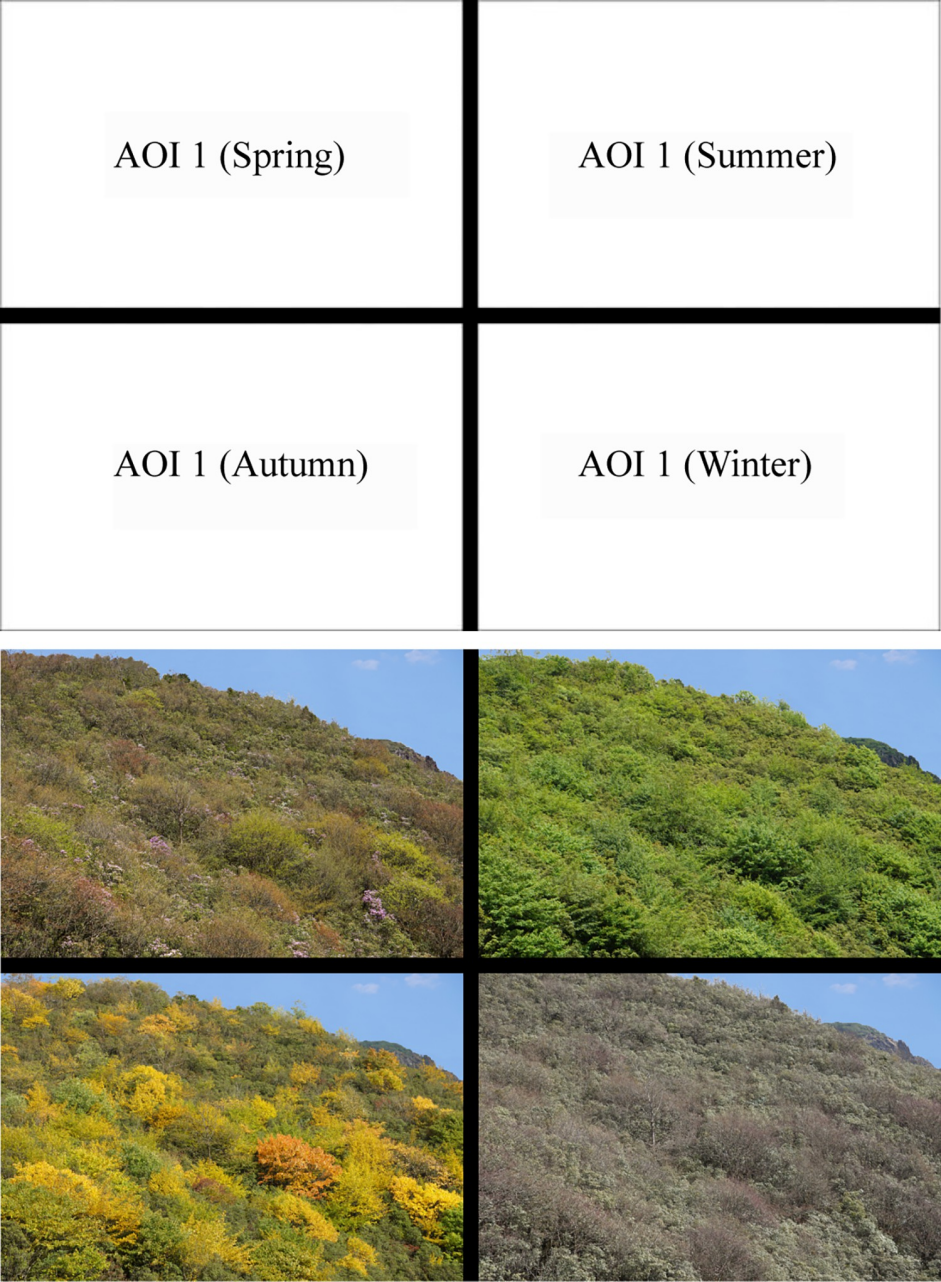
Contrast photos of forest color in the four seasons. **(A)** Before filling before filling. **(B)** Contrast pictures after filling.

In this study, the four seasons’ forest color photos of Jiaozi Mountain were spliced into a four-season contrast image of Jiaozi Mountain forest color. Each season’s contrast image was divided into four areas of interest, with the spring, summer, autumn, and winter forest color photos each corresponding to one area of interest (Fig 2). In addition, in order to avoid the preference impact of a different sky, we use Adobe Photoshop CC (2019, Adobe, San Jose, CA, United States) software to change the sky part of each four-season comparison picture into the same blue sky and white cloud. A total of 30 four-season comparative pictures were included in the experiment, including one preparation picture before the experiment and 29 formal photos of the experiment.

#### Experimental subjects

In terms of the selection of subjects, some studies have shown that there is no significant statistical difference in the aesthetic differences among people with different groups, different cultural backgrounds or even different working backgrounds [26]. It is feasible and representative to choose college students to conduct visual behavior experiment [27, 28]. In this study, 60 students and postgraduates from Southwest Forestry University were selected as the research objects. After the invalid samples were eliminated, the final sample consisted of 45 people (21 women, 24 men, mean age = 23, range = 21–26 years). Participants were recruited through a poster in August 2020. And then the specific time and place of the experiment were determined after the participant signed the informed consent. The experiment was conducted in a relatively independent classroom at Southwest Forestry University from September 1 to 4, 2020. The subjects were all students who voluntarily chose to take part in the experiment. Their natural or corrected visual acuity was above 1.0. In addition, relevant studies have shown that false eyelashes and glasses will have an impact on the acquisition of eye movement data, and eyes that are too small may also make the eye tracker unable to track the pupil of the subjects, resulting in an inability to collect data [29]. In order to avoid affecting the data acquired by eye movement devices, the subjects were required not to wear false eyelashes, to have normal color vision, and to have no eye problems such as color blindness, strabismus, amblyopia, and astigmatism [30].

#### Experimental equipment

A Tobii X2-30 non-contact eye tracker (Tobii Pro, Stockholm, Sweden) was used to record subjects’ visual behavior data with a sampling rate of 120 Hz. The experimental material (6000 x 4000 pixels) was displayed on a 24-inch display, the screen resolution was 1920 x 1080 pixels, and the eye tracking device was installed below the display. The display and data processing of experimental materials were controlled by the ErgoLAB human‒machine environment synchronization platform (KINGFAR, Beijing, China).

#### Experimental process

In order to avoid the interference of light, noise, and other external conditions with the visual behavior experiment, the experiment was carried out independently in a relatively closed room [31]. We adjusted the sitting position of the subjects, and kept the horizon, 60‒70 cm away from the screen. Then the subjects were briefed on necessary information such as the experimental process and requirements, but the purpose of the experiment was not explained so that the subjects could freely view the forest color photos according to their own preferences. After we were sure that the subjects understood the experimental requirements, they were calibrated to ensure the normal collection of eye movement data. After the eye movement calibration was completed, the experiment began. First, an experimental preparation photograph was shown to allow the subjects to enter the experimental state quickly. Subsequently, 29 seasonal contrast images of forest color were shown in a random order, with each image shown for 12 s. After the experimental material was shown, the eye movement data collection was stopped and the experiment was complete.

#### Visual behavior indicators

According to the theory of eye movement psychology, basic eye movement behaviors can be classified into three categories, including fixation, saccade and following movement [32]. In previous studies, it has been demonstrated that, it is difficult for the human visual system to process visual information in the process of saccade due to suppression. In the meantime, the following movement is excluded from analysis given that the eye movement test method adopted in this study is a static picture. Therefore, the visual behavior index of fixation form is selected as the main way to collect image information in study. At present, there are a relatively wide variety of indicators of fixation factors. To be exact, the indicators chosen for different research contents differ, including total fixation time, average fixation time, average fixation duration, total fixation number, and first fixation time, etc. [33, 34]. The three indicators including total fixation time, total fixation number and first fixation time are commonly used for prior studies. Combined with the AOI characteristics assigned by the study, these three indicators are adopted as the visual behavior indicators in this study. The cognition of these indicators is defined as follows:

AOI first fixation time (AFFT): the first time to reach the target area. Its unit is seconds (s). The shorter the first fixation time in the area of interest, the more attention the area of interest can attract.

AOI total fixation duration (ATFD): The total duration of attention in an area of interest, expressed in seconds (s). The longer the fixation duration, the more attractive the area of interest [35, 36].

AOI fixation count (AFC): Total number of fixations in an AOI; it usually reflects the familiarity of subjects with the region, and their interest in it. The higher the attention, the more important the region is for the subjects and the more attention it can attract [37].

### Landscape preference assessment

In a comparability study of indoor landscape preference evaluation and visual behavior, the same experimental samples are usually used [38, 39]. At the end of the visual behavior experiment, the subjects were asked to complete an image questionnaire survey on the color landscape of Jiaozi Mountain in a single season using the SBE survey scale. We asked them to rate their preferences on a scale of ‒3 to 3 (‒3 = not at all preferred; 3 = highly preferred). A total of 116 images of forest color in a single season were included in the assessment of landscape preference. In order to avoid a sequence effect, the images were shown in a random order, 5 s for each image. The whole experiment lasted about 10 min.

### Color quantification of landscape photos

#### Color component index

Based on existing research and the color characteristics of Jiaozi Mountain in four seasons, the proportion of 16 hues (H1‒16) was calculated by MATLAB (self-made program V1.0 of the research team) and nonuniform color quantification [4]. The data were further processed to eliminate colors with a proportion of color pixels less than 1%, and finally the number of colors (NC) of each image was calculated.

1. Hue Ratio (H): A total of 16 hue ratios (H1, H2, H3, …, H16), where N_Hi_ is the sum of pixels of the ith hue; n is the total number of pixels.
$$HPi=\frac{N_{Hi}}{n\times 100}$$ (1)
2. Number of Colors (NC): Among the 144 colors, the proportion of pixels is not less than 1%. H_a_ represents color, including H1‒H16; S_b_ represents saturation, including S_1_‒S_3_, three saturation intervals; V_c_ represents value, including V_1_‒V_3_, three value ranges.
$$NC=SUM(H_{a}S_{b}V_{c}),H_{a}S_{b}V_{c}>1\%$$ (2)

#### Spatial composition index of color patches

This study used ArcGIS (10.3, Esri, Redlands, CA, United States) software to sketch forest color patches. The sketch objects were forest color images (4000 x 6000 pixels) in mountainous slopes with a high pixel count. Therefore, the accuracy was controlled by visual interpretation. In order to minimize the visual error, the details of color patches were outlined by magnifying the landscape images. In the process of sketching, the degree of color discrimination was sketched under normal vision and the color similarities and differences were regarded as the main criteria for color patch division. Afterwards, the vector data were transformed into raster data and the spatial relationship of color patches was calculated by FRAGSTATS (v4.2.1, University of Massachusetts, Amherst, MA, United States).

According to the existing research results and the landscape characteristics of Jiaozi Mountain, the forest color patch index was selected and divided into five categories and eight subcategories (Table 1).

**Table 1:** Spatial composition index of color patches.

Color patch index		Shorthand	Implication
Color patch area index	Proportion of coniferous forest	AP	Reflects the coverage richness and dominance of color patches.
	Broad-leaved forest proportion	BP	
	Shrubwood proportion	SP	
	Grass proportion	GP	
Color patch density index	Color patch density	PD	Reflects the intensity of color patches, the greater the value, the denser the color landscape
Color landscape Shape index	Color landscape Shape index	LSI	Reflects the complexity of color patch shape: the greater the value, the higher the complexity of the color patch shape.
Color patch aggregation index	Color patch of cohesion index	COH	Reflects the connectivity between different color patches: the greater the value, the higher the connection between color patches.
Color patch diversity index	Shannon’s diversity index of color patch	SHDI	Reflects the uniformity and complexity of the distribution of different color patches: the greater the value, the richer the color patch type.

### Tree species interpretation

The on-site visual interpretation of target forests in various locations was performed using telescopes (Bosma Coywolf II10 x 50, Guangzhou). The detailed photos were taken of the plants whose tree species names could not be determined on the spot, the corresponding plant specimens were collected for identification through internal industry, while the tree species composition of various target forests was sorted out and recorded.

### Color interpretation of tree species composition

According to the detailed identification and interpretation of tree species in Jiaozi Mountain forest by field investigation and internal industry, the landscape patches of different tree species types were delineated using ArcGIS to build the patch distribution map comprised of tree species. The color difference of each tree with the season was obtained by overlaying the distribution map of tree species with the distribution map of color patches in different seasons. That is to say, tree species presents the color patches in different seasons. At the same time, the tree species patch distribution map was inputted into Matlab for extracting the color component index, and the hue information of each tree species was used to interpret the color characteristics of tree species composition.

### Analysis and statistics

This study used Daniel’s method to standardize the SBE value of each photo to reduce the aesthetic bias caused by individual differences [22]. At the same time, the ErgoLAB human‒machine environment synchronization platform (KINGFAR, Beijing, China) was used to process the original data of visual behavior. After data standardization, a further analysis was conducted using SPSS (26.0, IBM, Armonk, NY, United States) to explore the relationships between visual behavior and landscape preference, and between visual behavior and forest color characteristics. The specific analysis steps were as follows:

1. One-way analysis of variance (ANOVA) was used to analyze the differences of color characteristics between different seasons in Jiaozi Mountain.
2. The Pearson correlation analysis method was used to explore the relationships between forest color visual behavior and landscape preference, and between visual behavior, and forest color characteristics, in different seasons.

## Results

### Change of color characteristics in different seasons of Jiaozi mountain

#### Color characteristics

**Fig 3:**
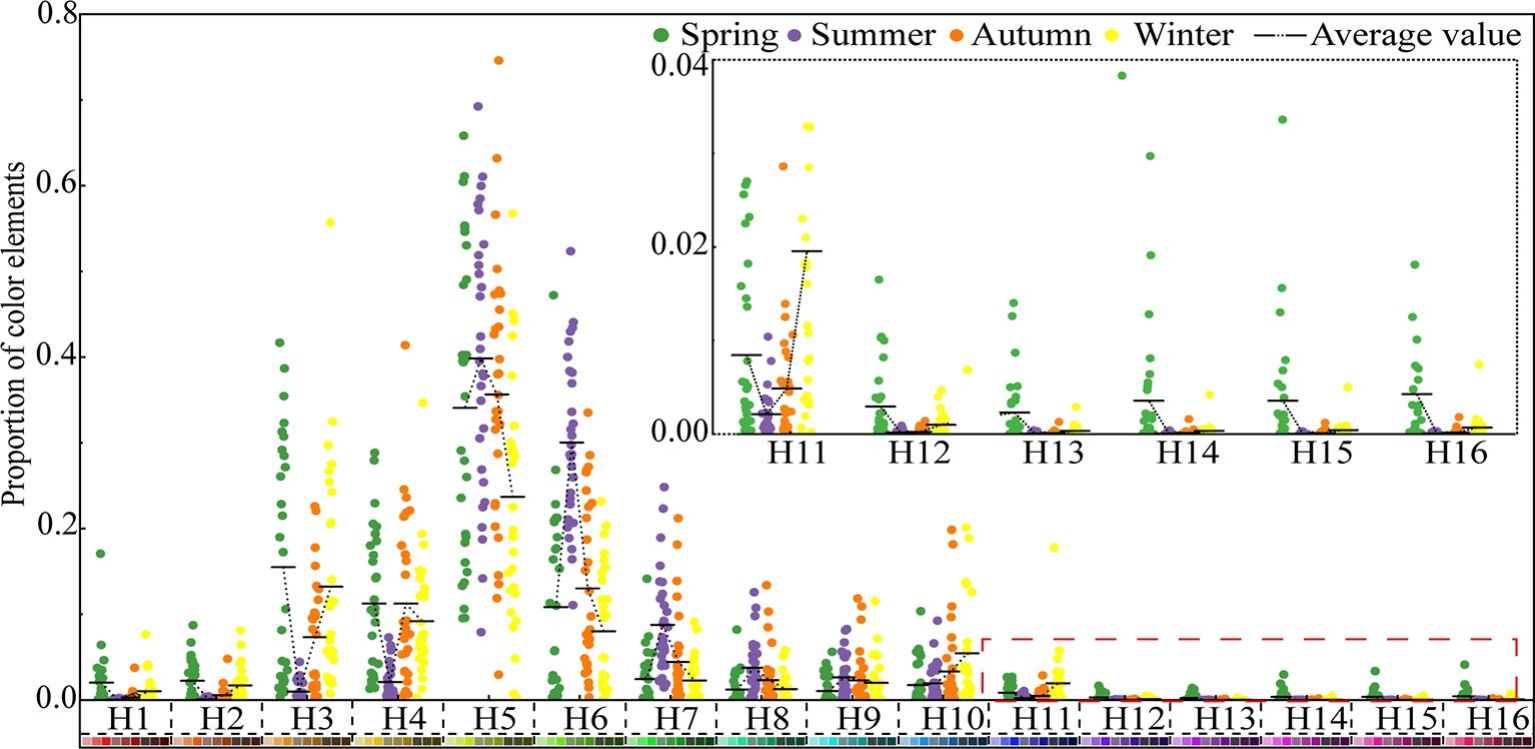
Proportion of color elements in spring, summer, autumn, and winter.

Fig 3 shows the proportion of hue elements in different seasons of Jiaozi Mountain in spring, summer, autumn, and winter. It can be clearly seen from the overall tone that the colors of the forest in Jiaozi Mountain are different in the four seasons, and the hues involved are red, yellow, green, blue, and blue-purple. In general, the forest of Jiaozi Mountain is mainly yellow (H3, H4), green (H5‒9) and blue (H10), while red (H1, H2), blue (H11) and blue-violet (H12‒16) are relatively less frequent. From the proportion of the four seasons with different colors, the color change degree of four seasons in Jiaozi Mountain is more significant. With the change of the four seasons’ phenology, plants show different seasonal characteristics, and the change of forest color in different seasons has obvious differences. In spring, the forest color of Jiaozi Mountain presents diversified forms, mainly red (H1, H2), yellow (H3, H4), green (H5–9), and blue (H10). Blue-violet (H12–16) is relatively scarce, but compared with summer, autumn, and winter, the blue-violet (H12–16) reaches the peak in spring. In summer, the forest color of Jiaozi Mountain is monotonous, mainly green (H5–H9), and other colors account for less. As the autumn progresses, the forest color richness of Jiaozi Mountain gradually increases, mainly with yellow (H3, H4), green (H5–9), and blue (H10). Red (H1, H2) and blue (H10) increase compared with the summer, but the proportion of these hues is still small. In winter, red (H1, H2), yellow (H3, H4), and blue (H10) increase gradually with seasonal changes. Green (H5–H9) reaches its lowest value in winter, but its color proportion remains dominant in winter. The winter colors of Jiaozi Mountain forest are mainly yellow (H3, H4), blue (H10), and green (H5–9).

Fig 3 shows the change trend of forest color hue ratio in different seasons. With the change of seasons, the proportions of hues of H1‒3 and H11‒16 first decreased and then increased. The proportions of hues reached the lowest value in summer. Except for hue H11, the other hues reached the highest value in spring. H5‒9 increased to a peak in summer, and then decreased. H10 continued to increase with the seasonal variation, while H4 decreased at first, then increased and finally decreased with the seasonal variation, reaching its lowest value in summer. The proportion of hues tended to be consistent and reach the highest value in the spring and autumn.

One-way analysis of variance (ANOVA) was performed on forest hue ratios in different seasons. The results showed that, except for hue H9, there was no significant difference between the different seasons (P > 0.05), while other forest hue factors were significant (P < 0.05) or extremely significant (P < 0.01). Multiple comparison tests showed that hues H1 and H12‒16 showed no significant difference in summer, autumn, and winter, but they were significantly different from those in spring. H4, H6, H7, and H8 did not change significantly in spring, autumn, and winter, but changed significantly in summer. H5 and H11 showed no significant difference in spring, summer, and autumn, but had a significant difference in the winter. H2 showed a significant difference between spring and winter, summer, and autumn, but no significant difference between other seasons. There was no significant change in H3 between spring and summer, but there were significant differences between these two seasons and the other seasons. There was no significant difference in H10 between spring, summer, and autumn; there was a significant difference in winter compared with spring and summer, but no significant difference with autumn (Table 2).

**Table 2:** Proportion and difference of forest color composition in different seasons.

Hue	Hue proportion in different seasons (%)				F	P
	Spring	Summer	Autumn	Winter		
H1	2.03 ± 3.35a	0.05 ± 0.07b	0.30 ± 0.70b	1.03 ± 1.62b	6.373	0.001
H2	2.25 ± 2.38a	0.08 ± 0.12b	0.57 ± 0.93b	1.71 ± 1.97a	11.138	<0.001
H3	15.50 ± 13.97a	0.08 ± 1.15c	7.34 ± 6.62b	13.21 ± 12.69a	12.209	<0.001
H4	11.24 ± 8.43a	2.09 ± 2.12b	11.25 ± 9.87a	9.20 ± 6.93a	9.902	<0.001
H5	34.09 ± 17.86a	39.84 ± 15.84a	35.64 ± 15.97a	23.69 ± 14.50b	5.276	0.002
H6	10.84 ± 11.43b	30.04 ± 9.89a	13.02 ± 9.98b	8.00 ± 7.19b	30.090	<0.001
H7	2.43 ± 3.19b	8.76 ± 6.03a	4.45 ± 5.52b	2.26 ± 2.48b	12.759	<0.001
H8	1.21 ± 1.82b	3.75 ± 3.18a	2.31 ± 3.31b	1.26 ± 1.46b	6.228	0.001
H9	1.04 ± 1.54b	2.63 ± 2.42a	2.30 ± 3.27ab	2.01 ± 2.66ab	2.092	0.105
H10	1.74 ± 2.60b	1.93 ± 2.15b	3.31 ± 5.24ab	5.46 ± 8.58a	3.031	0.032
H11	0.84 ± 0.91b	0.21 ± 0.24b	0.49 ± 0.61b	1.95 ± 3.37a	5.391	0.002
H12	0.30 ± 0.42a	0.02 ± 0.02b	0.03 ± 0.03b	0.10 ± 0.11b	10.355	<0.001
H13	0.23 ± 0.37a	0.01 ± 0.01b	0.01 ± 0.02b	0.03 ± 0.06b	9.565	<0.001
H14	0.36 ± 0.67a	0.01 ± 0.01b	0.01 ± 0.03b	0.04 ± 0.08b	7.460	<0.001
H15	0.36 ± 0.70a	0.01 ± 0.01b	0.01 ± 0.02b	0.04 ± 0.09b	6.681	<0.001
H16	0.43 ± 0.84a	0.01 ± 0.01b	0.02 ± 0.04b	0.07 ± 0.12b	6.457	<0.001

Different letters indicate significant differences at the 0.05 level.

In addition, the variance analysis of the number of colors (NC) in different seasons showed that the number of colors (NC) was significantly different in different seasons (P < 0.01). The four seasonal indexes are 19.45, 17.38, 17.76, and 16.14, respectively, reaching the peak in spring, showing a trend of first decreasing, then increasing, and finally decreasing.

#### Characteristics of color patches

By comprehensive comparison of the change of color patch index with season in Jiaozi Mountain forest landscape, it can be seen that there are significant differences in other indicators besides the color patch area index, which changes with the seasons. The color patch density (PD) and color landscape shape index (LSI) changed significantly in different seasons, reaching the highest value in spring and the lowest value in summer. There was no significant difference between summer, autumn, and winter, but the difference was significant in spring. The color patch of cohesion index (COH) reached the lowest value in spring, and the change was not obvious in summer, autumn, and winter. The Shannon’s diversity index of color patch (SHDI) was significantly different between spring and winter, but there was no significant difference between summer and autumn (Table 3).

**Table 3:** Difference analysis of forest color patches in different seasons.

Color patch index	Proportion of color patches in different seasons (%)				F	P
	Spring	Summer	Autumn	Winter		
PD	3.18 ± 2.02a	1.03 ± 0.76b	1.71 ± 1.17b	1.17 ± 1.09b	15.525	<0.001
LSI	9.96 ± 3.86a	4.86 ± 1.57b	5.78 ± 1.97b	4.90 ± 3.29b	26.183	<0.001
COH	99.94 ± 0.03b	99.96 ± 0.02a	99.96 ± 0.02a	99.97 ± 0.02a	9.743	<0.001
SHDI	1.56 ± 0.46a	1.34 ± 0.53ab	1.33 ± 0.50ab	1.15 ± 0.51b	3.326	0.022

Different letters indicate significant differences at the 0.05 level.

### Evaluation of forest color preference in Jiaozi mountain

**Fig 4:**
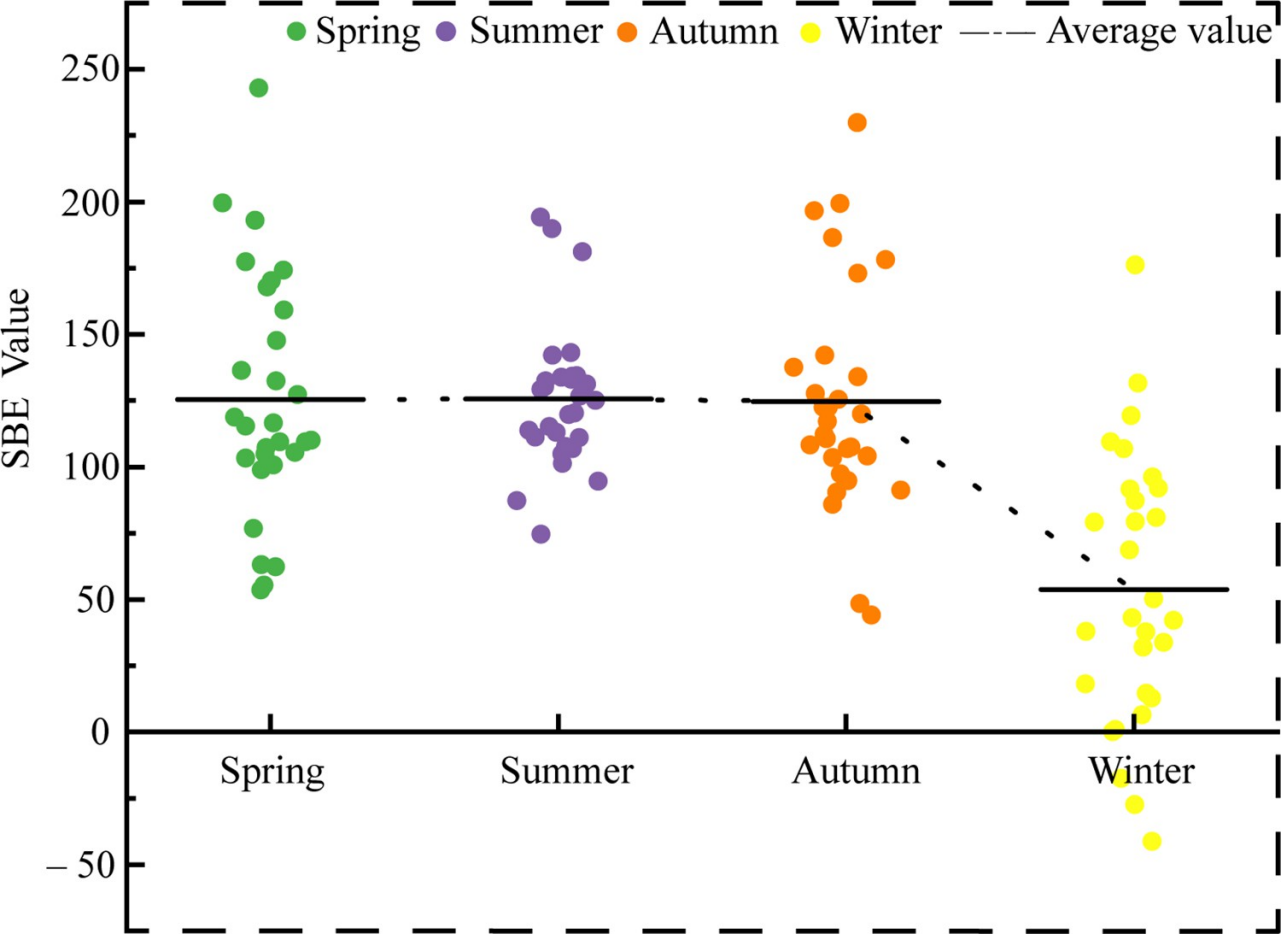
Variation trend of SBE value in different seasons of Jiaozi Mountain forest.

Fig 4 shows the variation trend of SBE values in Jiaozi Mountain forest in different seasons. In the overall beauty analysis, the beauty value range of all color landscape photos in four seasons of Jiaozi Mountain forest was ‒41.28 to 242.91, with an average value of 107.39. With the change of seasons, the change trend of beauty value was not obvious in spring, summer, and autumn, but it decreased suddenly in winter. The average values of the four seasons were 125.47, 125.57, 124.71, and 53.82, respectively. There was no significant difference in the values of the four seasons in spring, summer, and autumn, but they were lowest in winter, and significantly different from those in spring, summer, and autumn.

### Correlation between visual behavior and landscape preference

#### The correlation between visual behavior and landscape preference in the comparison of four seasons’ photos

The relationship between visual behavior and landscape preference is shown in Table 4. The visual behavior indexes AOI first fixation time (AFFT), AOI total fixation duration (ATFD), and AOI fixation count (AFC) were significantly correlated with the subjective evaluation indexes SBE values, among which AOI first fixation time (AFFT) was significantly negatively correlated with SBE values. AOI total fixation duration (ATFD) and AOI fixation count (AFC) were highly significantly positively correlated with SBE value, and the correlation coefficient was ranked as AOI total fixation duration (ATFD) > AOI fixation count (AFC), from high to low.

**Table 4:** Pearson correlation coefficient between visual behavior and landscape preference.

	Landscape preference
AOI first fixation time	‒0.402**
AOI total fixation duration	0.611**
AOI fixation count	0.593**

** denotes *p* < 0.01

* denotes *p* < 0.05.

#### The correlation between visual behavior and landscape preferences of forest colors of different seasonal types

Table 5 shows the correlation between visual behavior and landscape preference of different seasonal types. For forest colors in spring, autumn, and winter, AOI total fixation duration (ATFD) and AOI fixation count (AFC) were significantly positively correlated with SBE value, while AOI first fixation time (AFFT) was not significantly correlated with SBE value. There was no significant correlation between the SBE value of forest color in summer and any visual behavior index.

**Table 5:** Pearson correlation coefficient between visual behavior and landscape preference for forest color in different seasons.

	Spring	Summer	Autumn	Winter
AOI first fixation time	‒0.091	‒0.050	‒0.168	‒0.280
AOI total fixation duration	0.791**	0.125	0.429*	0.427*
AOI fixation count	0.775**	0.136	0.379*	0.498**

** denotes *p* < 0.01

* denotes *p* < 0.05.

### The correlation between forest color characteristics and visual behavior of forest color photos in different seasons

#### The correlation between forest color features and visual behavior in the four seasons

**Fig 5:**
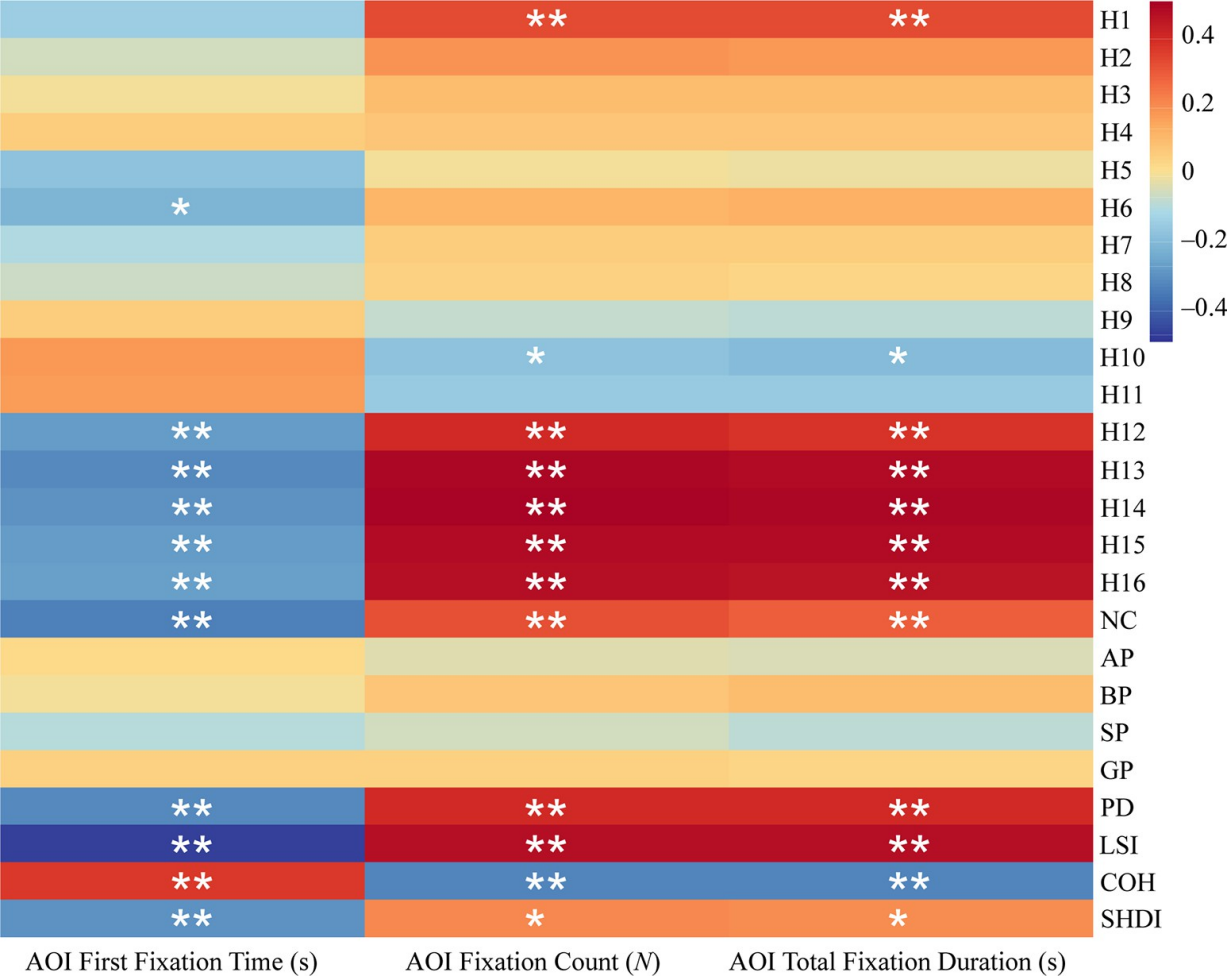
Correlation between color characteristics and visual behavior of forest color photos in all seasons. ** denotes *p* < 0.01, * denotes *p* < 0.05.

It can be seen from Fig 5 that the visual behavior indexes of participants were significantly correlated with many forest color characteristics indexes. In terms of forest color components, AOI first fixation time (AFFT) was significantly (P < 0.05) or extremely significantly negatively correlated with green (H6), blue-violet (H12‒16), and the number of colors (NC) (P < 0.01). The visual behavior indexes AOI total fixation duration (ATFD) and AOI fixation count (AFC) were significantly positively correlated with red (H1), blue-violet (H12‒16), and the number of colors (NC), but significantly negatively correlated with blue (H10). The color components of other indicators had no significant relationship with visual behavior indicators (P > 0.05).

Among the forest color patch composition indexes, color patch density (PD), color landscape shape index (LSI), and Shannon’s diversity index of color patch (SHDI) were significantly (P < 0.05) or extremely significantly negatively correlated with AOI first fixation time (AFFT) (P < 0.01), and significantly (P < 0.05) or extremely significantly positively correlated with AOI total fixation duration (ATFD) and AOI fixation count (AFC) (P < 0.01). On the contrary, the color patch binding index (COH) had a positive correlation with AOI first fixation time (AFFT), and a negative correlation with AOI total fixation duration (ATFD) and AOI fixation count (AFD) (P < 0.01).

### The correlation between color characteristics and visual behavior of forest color photos in different seasons

**Fig 6:**
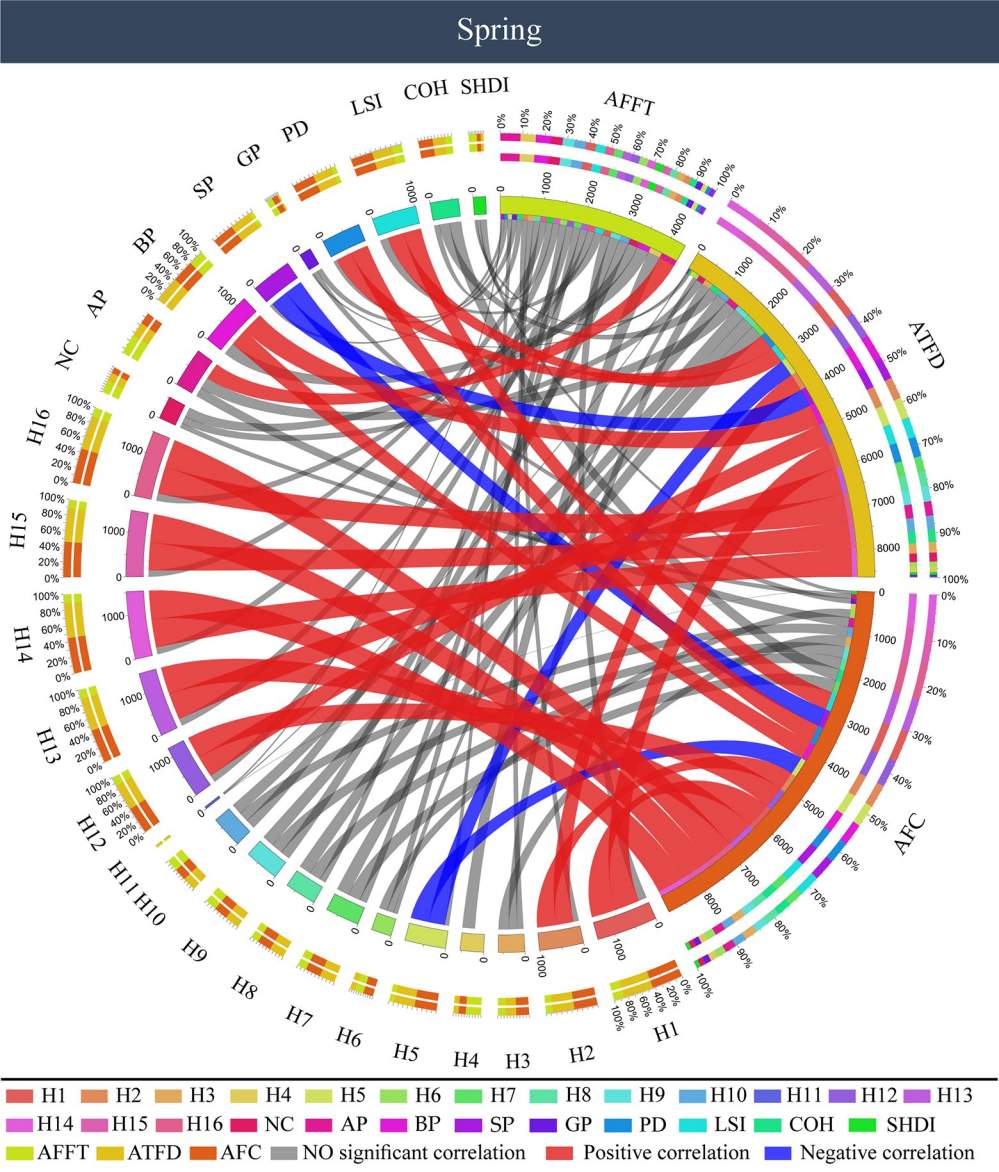
Correlation between color characteristics and visual behavior of forest color photos in spring. AFFT: AOI first fixation time, ATFD: AOI total fixation duration, AFC: AOI fixation count.

Fig 6 gives the results of a correlation analysis between the color characteristics and visual behavior of forest color photos in different seasons. For a spring forest color, AOI first fixation time (AFFT) was significantly positively correlated with the proportion of coniferous forest color patches (AP) (P < 0.05); there was no significant correlation with forest color components and other forest color patches (P > 0.05). The correlation results between the AOI total fixation duration (ATFD) and forest color characteristic indexes were consistent with those between the AOI fixation count (AFC) and forest color characteristic indexes. Among the forest color components, AOI total fixation duration (ATFD) and AOI fixation count (AFC) were positively correlated with red (H1, H2) and blue-violet (H12‒16), and negatively correlated with green (H5) (P < 0.05). Among the spatial components of forest color patches, the two are significantly positively correlated with the proportion of broad-leaved forest color patches (BP), color patch density (PD), and color landscape shape index (LSI), and negatively correlated with the proportion of shrubwood (SP) (P < 0.05).

**Fig 7:**
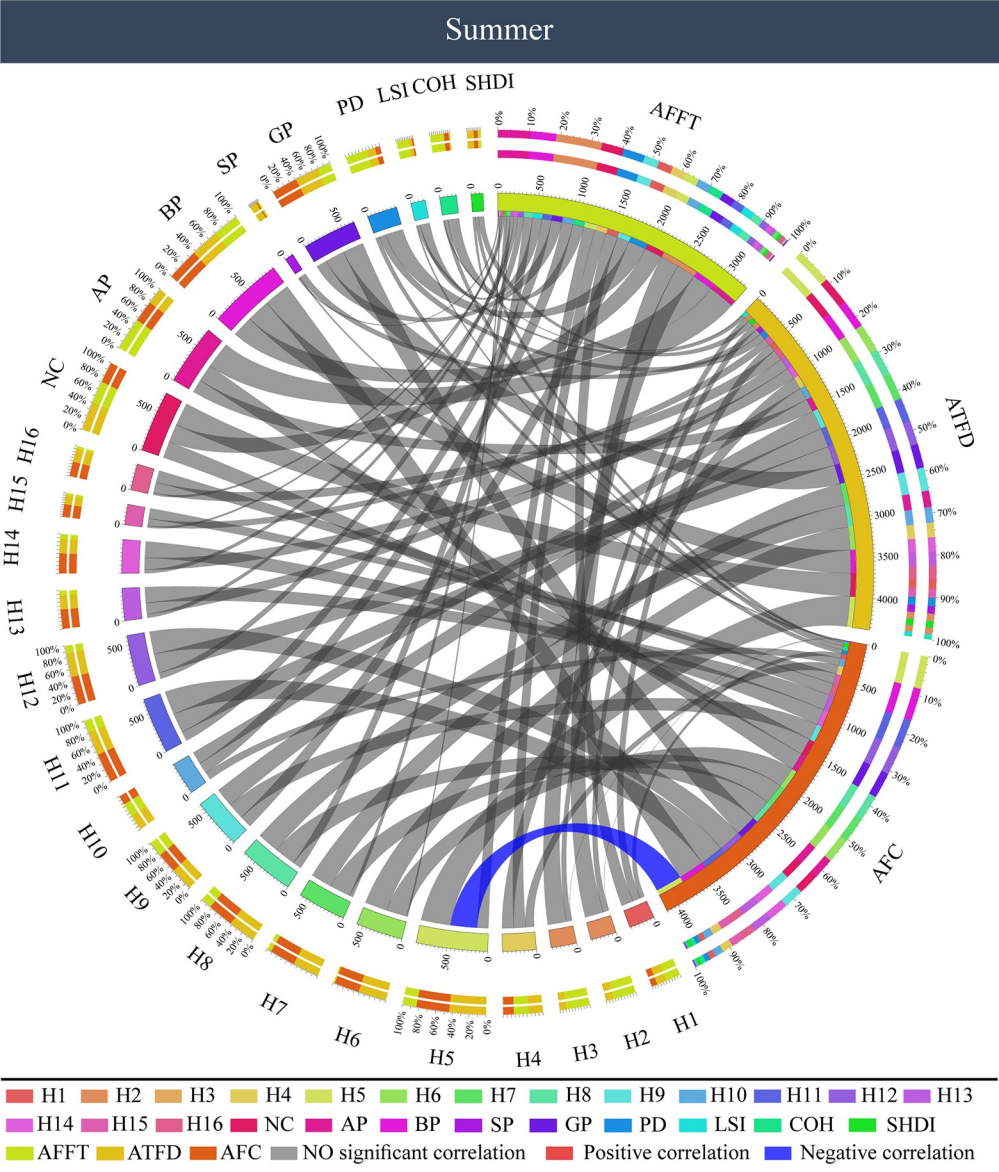
Correlation between color characteristics and visual behavior of forest color pictures in summer. AFFT: AOI first fixation time, ATFD: AOI total fixation duration, AFC: AOI fixation count.

In a summer forest color (Fig 7), AOI total fixation duration (ATFD) was significantly negatively correlated with green (H5) (P < 0.05). The more H5 in color landscape photos, the less total attention subjects gave to the summer forest color. Other visual behavior indicators, AOI first fixation time (AFFT) and AOI fixation count (AFC), were not significantly correlated with forest color characteristics (P > 0.05).

**Fig 8:**
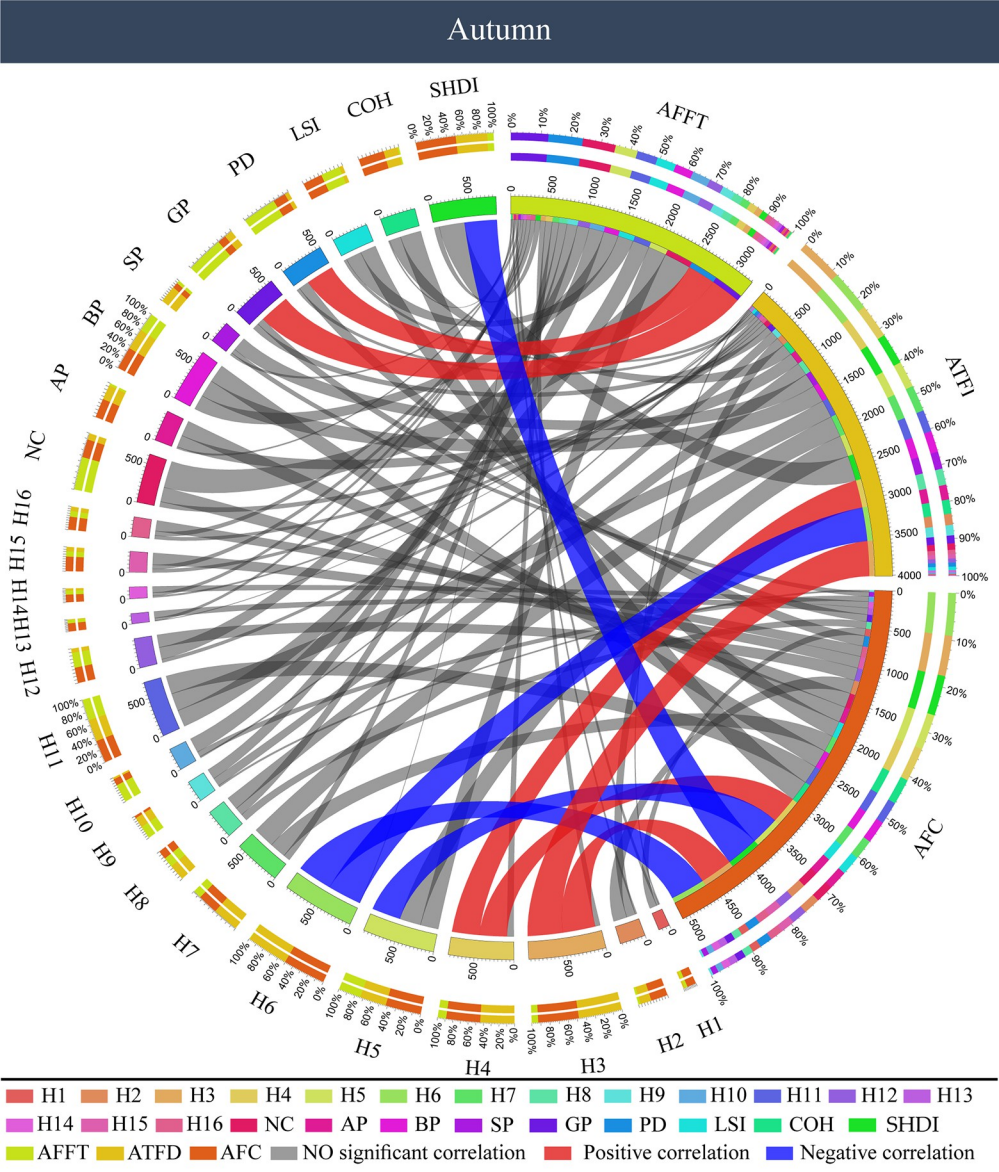
Correlation between color characteristics and visual behavior of forest color pictures in autumn. AFFT: AOI first fixation time, ATFD: AOI total fixation duration, AFC: AOI fixation count.

As to the autumn forest color, a Pearson correlation analysis showed that (Fig 8) AOI first fixation time (AFFT) was positively correlated with the proportion of grassland color patches (GP) and color patch density (PD) (P < 0.05), but had no correlation with forest color components (P > 0.05). On the contrary, AOI total fixation duration (ATFD) is only correlated with the indicators of forest color components, but has no significant correlation with the color patch components. For color components, AOI total fixation duration (ATFD) was positively correlated with yellow (H3, H4) and negatively correlated with green (H6) (P < 0.05). AOI fixation count (AFC) has a significant correlation with color components and color patch components. It is positively correlated with yellow (H3, H4), and negatively correlated with green (H5, H6) (P < 0.05). Among the color patch components, AOI fixation count (AFC) was only negatively correlated with the Shannon’s diversity index of color patch (SHDI) (P < 0.05).

**Fig 9:**
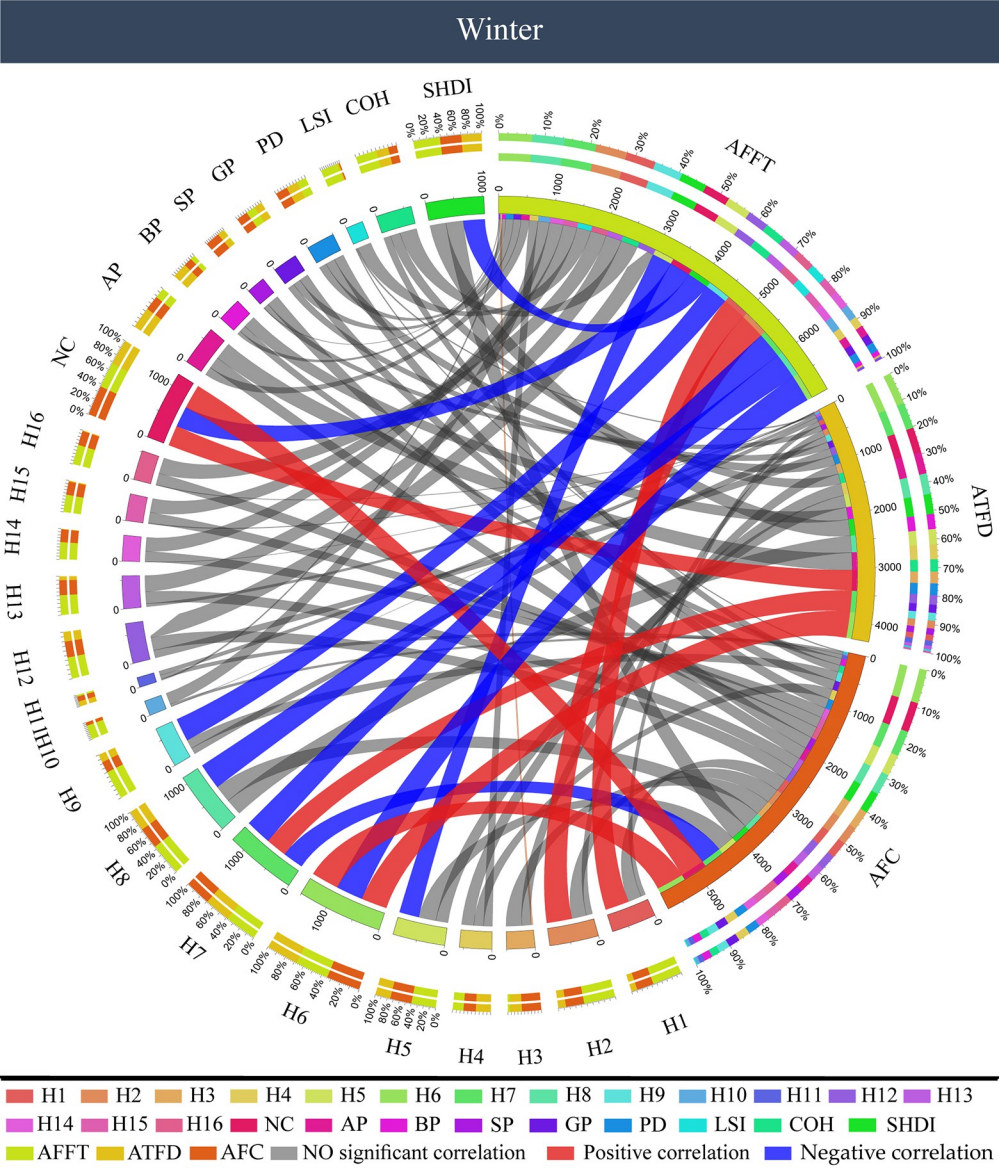
Correlation between color characteristics and visual behavior of forest color pictures in winter. AFFT: AOI first fixation time, ATFD: AOI total fixation duration, AFC: AOI fixation count.

According to the correlation analysis results of color characteristics and visual behavior of winter forest color photos (Fig 9), among the color components, AOI first fixation time (AFFT) had a significantly positive correlation with red (H1, H2), and a significantly negative correlation with green (H5‒9) and the number of colors (NC) (P < 0.05). For the visual behavior indicators AOI total fixation duration (ATFD) and AOI fixation count (AFC), a higher proportion of green (H6, H7) and a larger number of colors (NC) led to higher total fixation duration and fixation times. As to the components of color patches, AOI first fixation time (AFFT) was negatively correlated with the Shannon’s diversity index of color patch (SHDI) (P < 0.05). AOI total fixation duration (ATFD) and AOI fixation count (AFC) had no correlation with color patch components (P > 0.05).

## Discussion

### Color characteristics of four seasons in Jiaozi Mountain

In general, the color change in forest landscapes across the four seasons of Jiaozi Mountain is significant. The proportion of hues in different seasons is different, and the color changes are significantly different. The color is the most abundant in spring and autumn, while the color richness is relatively low in summer and winter. The spring tone of the forest in Jiaozi Mountain mainly include red (H1, H2), yellow (H3, H4), green (H5‒9), and blue (H10, H11). The blue-violet (H12‒16) accounts for a small proportion. However, compared with the other three seasons, the blue-violet system is relatively large in spring, and the proportion is relatively small or tends to be absent in the rest of the seasons. The main hue of the forest in the summer is green (H5‒9), and the proportion of other color systems decreases significantly. The proportion of red (H1, H2) and yellow (H3, H4) is lowest in the summer. In autumn and winter, the green series decreases significantly and reached the lowest value in winter, while the red (H1, H2), yellow (H3, H4), and blue (H10, H11) increase significantly in autumn and winter.

The seasonal color changes in Jiaozi Mountain forest are consistent with its plant species and seasonal characteristics. Previous studies have shown that the forest itself conveys different colors based on the differences in plant physiological characteristics [40]. Plants change their phenology regularly with the change of climate in the four seasons, which makes the whole forest landscape show different colors in different seasons. The plant community of Jiaozi Mountain is mainly composed of evergreen trees, broad-leaved forest, and deciduous broad-leaved forest species such as *Pinus*, *Quercus*, *Acer*, *Populus*, and *Rhododendron*. Through color interpretation, the color characteristics of Jiaozi Mountain forest during different seasons are factored into the composition of tree species. In spring, plants such as *Rhododendron delavayi*, *Rhododendron lapponicum*, and *Rhododendron sphaeroblastum* blossom, mainly presenting red and blue-violet colors, which is also why red and blue-violet are more abundant in spring in Jiaozi Mountain than in the other three seasons. Some deciduous broad-leaved tree species, such as *Populus davidiana*, have young leaves, which are mainly yellow, grow in the spring. In autumn and winter, most green-leaf plants show discoloration and withering because of phenological changes as the chlorophyll inside the leaf is reduced [41]. Some deciduous broad-leaved species, such as *Acer* and *Castanea mollissima*, have yellow and red leaves in the autumn and winter, resulting in an increase in the proportion of yellow and red in the autumn and winter, and a decrease in the proportion of green. Evergreen coniferous forests and broad-leaved forest plants such as *Pinus yunnanensis*, *Pinus armandii*, and *Quercus pannosa* are green in multiple seasons. Therefore, the green of the forest in Jiaozi Mountain occupies a large proportion in the four seasons. The number of colors (NC), affected by plant discoloration, varied significantly in different seasons. Plant color reached peak richness with the phenological changes in the spring and autumn; the number of colors (NC), consistent with this trend, peaked in spring and autumn. The number of colors (NC) is lower in summer and winter, but they are different. Summer color is mainly green, while other color systems account for less. In winter, the forest color of Jiaozi Mountain has different proportions. In terms of color diversity, compared with summer, spring, autumn, and winter have higher color diversity, and the summer forest color is monotonous.

As for the color patch index, except that there is no significant difference in the color patch area index with the seasonal variation, the other indicators are significantly different. This shows that, in addition to the proportion of different plant patches, the spatial composition and layout of color patches show significant differences over time. There was no significant plant change in Jiaozi Mountain from May 2019 to August 2020. Therefore, the area of color patches of coniferous forest, broad-leaved forest, shrub forest, grassland, and other plants did not change significantly during this period. Studies have shown that plant community composition is the main factor determining the spatial distribution of forest color patches [42]. The spatial distribution of color patches is mainly affected by the composition of forest color, and the spatial distribution of color patches in different seasons is consistent with the color change of plant communities. There are obvious differences in the composition of plants in different areas of Jiaozi Mountain forest, and the same species grow in aggregation. In spring, the color richness of Jiaozi Mountain forest is high and diversified, which leads to the peak density of color patches in spring and the complex and diverse shape of the color landscape. In addition, the same plant species in the same season showed consistent colors, so the color patch of cohesion index (COH) was high. The number of colors of the forest in Jiaozi Mountain was highest in spring and lowest in winter. The higher the color richness, the richer the color patch types. Therefore, in spring, the forest color patch in Jiaozi Mountain was the most abundant and the color patch diversity was high. The color patch type is the most monotonous in winter.

### Forest color preference evaluation

The beauty degree of the forest color is different in different seasons. There was no significant difference in the beauty degree in spring, summer, and autumn, but the difference of beauty degree in winter (the lowest value) was significant. This means that the subjects’ overall preference for the forest color varies from season to season. Compared with the forest color in winter, the beauty degree of the forest color in Jiaozi Mountain in spring, summer, and autumn was higher, so the subjects preferred the forest color in these three seasons. In spring, some of the flowering plants gradually bloom, and the forest becomes colorful. In summer, most plants appear green, and the forest color is ever-green. In autumn, the color of leaves and other plant organs of some deciduous broad-leaved tree species changes. At this time, yellow is more common and the forest gets more colorful. In winter, the leaves of some plants began to wither as the phenology varied, which was accompanied by the forest color changes. Moreover, the forest color achieved the lowest amount, and the respective color index accounted for a relatively low proportion. In addition, influenced by the color composition of plants, the Shannon’s diversity index of Color Patch (SHDI) reached its lowest value in winter, and color patches tended to be homogenized. Furthermore, the color richness was lower than that of the other three seasons. Single color was found to be easy to cause visual fatigue of observers, and with the increase in the color amount and the patches richness, the aesthetic effect of the landscape was improved. The findings above were consistent with existing studies that the amount of colors and the composition and proportion of colors are vital factors of the aesthetic effect of forest landscape. Based on a wide variety of color attributes, different ornamental effects could be generated. At the same time, under the same or similar color amounts and attributes, a range of spatial patterns exerted different aesthetic effects. Significant and extremely significant correlations were identified between SBE value, color characteristic index and color pattern index. Besides, significant and extremely significant positive correlations were reported between the amount of colors, the Shannon’s diversity index of Color Patch (SHDI) and SBE value. When the amount of colors increased, the higher the color diversity and richness, the higher the aesthetics of the landscape would be [43, 44].

### The correlation between visual behavior, landscape preference, and color characteristics of forest color

#### The correlation between forest color visual behavior and landscape preference, color characteristics in all seasons

Studies have shown that visual behavior is the response of human beings to their own psychological cognition and physiological processes [3]. Human perception of forest landscape is a process from local recognition and analysis of landscape features to overall preference cognition [1]. In the early stage of perception, visual behavior is mainly driven by bottom-up visual guidance mechanisms [45, 46], then gradually replaced by top-down visual guidance mechanisms [47]. This study investigated the correlation between visual behavior of forest color and landscape preference, visual behavior, and color characteristics in all seasons. The research found that participants’ visual behavior indicators AOI first fixation time (AFFT), AOI total fixation duration (ATFD), and AOI fixation count (AFC) were significantly correlated with SBE values. This is consistent with the study of Huang. There is a certain correlation between the visual behavior of forest landscape and landscape preference. The visual behavior index can predict the preference of subjects for a landscape to a certain extent [37]. When the forest color is presented in the form of four-season contrast images, the contrast images provide a more complete vision of the forest color. The subjects judge the pictures according to other images appearing in the pictures, identify the landscape pictures earlier, and produce a cognitive preference evaluation. Compared with the forest color in a single season, the four-season comparison images may be easier to identify and recognize. Therefore, whether in the early or late cognitive recognition process, subjects’ visual behavior indicators have a certain correlation with landscape preferences. The correlation between AOI first fixation time (AFFT), AOI total fixation duration (ATFD), AOI fixation count (AFC), and landscape preference can be explained by the fact that, the shorter the first glimpse the subject has at an area of interest, the higher the total number of gazes at the area of interest, and the longer the total gaze time, the higher the visual attractiveness of the area of interest and the higher the preference of the subject for this area. In terms of the correlation between visual behavior and color characteristics, we found that visual behavior indicators were significantly correlated with several forest color indicators. Green, red, and blue-violet accounted for more, while blue accounted for less; the color quantity was rich and the color patch heterogeneity was high; the patch fragmentation degree of the forest color interest area meant that the higher the degree of attention, the higher the visual attractiveness of the interest area, and the higher the preference of subjects for the area of interest.

#### The correlation between the visual behavior of forest colors in different seasons and landscape preference, color characteristics

Through a study of the correlation between the visual behavior of forest colors in different seasons and landscape color preferences, it was found that when the subjects observed the forest landscape in different seasons, the correlation between landscape preference, color characteristics and visual behavior was different. This is consistent with previous studies, which found that, when subjects observed different types of landscape, the relationship between visual behavior indicators and landscape preferences was not consistent. Our study found that there was consistency between the visual behavior of forest colors and landscape color preferences, in spring, autumn, and winter, but not in the summer.

In spring, autumn, and winter forest landscapes, the relationship between visual behavior indicators and color characteristics varies significantly. Different seasons of forest color attraction is different. The visual focus and visual attention of the subjects varies with the color information contained in the forest landscape. In spring, AOI first fixation time (AFFT) was positively correlated with the proportion of coniferous color patches (AP). That is to say, the smaller the proportion of coniferous color patches (AP), the less time subjects spend looking at the spring color landscape for the first time, and the more the area of interest could attract the attention of subjects. In autumn, AOI first fixation time was positively correlated with the proportion of grassland color patches (GP) and the density of color patches (PD). This shows that, when the proportion of grassland color patches and the density of color patches in an autumn forest landscape are higher, subjects take a longer first look at the autumn forest color. In winter, AOI first fixation time (AFFT) was negatively correlated with green (H5‒9) and the number of colors (NC), and positively correlated with red (H1, H2). This means that the greater the green color ratio, the higher the landscape attractiveness, while with the red of a larger winter color forest landscape it is less easy to attract the attention of participants. At the same time, AOI first fixation time (AFFT) was not correlated with the SBE value in these three seasons, indicating that subjects did not form a preference cognition for forest color photos when they first viewed them. The visual attention of subjects was mainly guided by relatively significant low-level visual attention. Previous studies have shown that visual behavior is related to the content of the image, and different content will greatly affect the subjects’ observation mode. Compared with other regions of the image, subjects would focus on the most salient regions earlier.

With regard to the visual behavior indicators, AOI total fixation duration (ATFD) and AOI fixation count (AFC), we found that both were significantly positively correlated with the SBE value, and there was a consistency between visual attractiveness and the overall preference for forest color. At the same time, the subjects observed the forest color in different seasons, so the relationship between visual behavior indicators and color characteristics was not consistent. About the spring forest color, the more heterogeneous the color patches, the richer the colors, and the higher the proportion of red and blue-violet, the more subjects’ fixation points and the higher their total fixation time, the higher their visual attraction, and the higher their preference. In autumn forest color, yellow and green significantly affect the visual behavior of subjects. The more yellow there is, the less green there is, and the greater the total fixation time of subjects. In addition, the diversity of color patches has a certain impact on subjects’ attention. The higher the diversity index of color patches, the higher the heterogeneity of patches, the greater the subjects’ attention, and the higher the degree of preference In winter forest color, subjects’ total fixation time was significantly higher when the number of colors was higher and when green occupied a higher proportion, and decreased with the decrease of the two factors. This shows that the green color richness and color quantity of a winter forest color make the visual attractiveness higher, which means that subjects prefer this kind of winter forest color. In addition, we found that, among the beauty values given by the subjects, there were significant differences between spring, autumn, and winter; subjects preferred the forest color in spring and autumn. Although the landscape preference results of spring, autumn, and winter forest color were quite different, there was consistency between the visual attractiveness of the forest color and it being preferred overall in these three seasons. The reason may be that, although there is a certain correlation between the visual behavior of subjects in terms of the forest color and the beauty value, they are still two relatively independent indicators in general. Landscape preference evaluation is more to evaluate the overall beauty of the evaluated object. The visual behavior law represents the visual acquisition and attention process of participants in terms of the color information of forest landscape, and pays more attention to the richness of color information of forest landscape and the attractiveness of the forest color, which is different from the beauty value. Spring, autumn, and winter, due to the diversity of the color landscape and the rich visual attention, formed a more consistent relationship.

The summer forest color was significantly different in terms of the visual behavior and landscape preferences. In terms of visual behavior and landscape preference, there was no significant correlation between the visual behavior index and SBE value. There was a certain difference between the overall preference and visual attractiveness of the forest color in summer. In the observation of a summer forest color, subjects assigned a higher beauty value (average: 125.57). In terms of visual behavior and color characteristics, apart from AOI total fixation duration (ATFD) being negatively correlated with green H5, there was no significant correlation between other visual behavior indexes and color characteristics. This shows that the subjects have a high overall preference for the summer forest color. In terms of the visual attractiveness of forest color, color information elements have no significant effect on the visual behavior of subjects. Compared with the other three seasons, the summer tone of the Jiaozi Mountain forest is given priority with its green system, low color richness, and relatively monotonous forest color. There is no interesting object in the visual field of the forest color. After observing the whole landscape, the subjects found that there was no color element with visual attractiveness, which led to a weak correlation between visual behavior and the color characteristics of the subjects. Therefore, this causes a difference between the overall preference and the visual attractiveness of the forest color in summer.

### Tree species regulation based on visual behavior

Through the above research, we found that landscape preference can be interpreted to a certain extent. While on the whole, there remain two relatively independent indicators. The evaluation of landscape preference is largely dependent on the overall beauty of forest color. As for visual behavior, it relates mainly to the visual attractiveness of forest colors. When the forest colors are observed by the subjects in different seasons, the color characteristic factors that could influence the visual behavior indexes differ as well. Therefore, when it comes to the planning and management of forest color, visual behavior experiments can be conducted as a means of quantitative research on forest color. Depending on the exact season, the forest color elements that could have impact on the visual behavior indicators can be applied to the composition of tree species for enhancing the visual attractiveness of forest color. In terms of forest management, forest ecological restoration and urban forest greening, we can use thinning and replanting according to different geographical and ecological conditions to replace some poorly growing tree species with seasonally changing tree species. In the process of urban barren hills and wastelands construction, ecological restoration, urban landscape improvement, and forest renewal, color patterns favored by the public can be used to configure the composition of tree species and spatial reinterpretation.

In spring, AOI first fixation time (AFFT) was positively associated with the Proportion of coniferous forest (AP), to a significant extent, which means that the smaller the proportion of coniferous forest, the shorter the time spent by the subjects observing the area of interest for the first time. Reducing the proportion of coniferous tree species, such as *Pinus yunnanensis* and *Pinus armandii*, is an effective solution to enhancing the visual salience of forest color. The color characteristic indexes including red (H1, H2), blue-violet (H12‒16), broad-leaved forest proportion (BP), color patch density (PD), color landscape shape index (LSI) and Shrubwood proportion (SP) are closely related to the visual behavior indexes AOI total fixation duration (ATFD) and AOI fixation count (AFC). This is because the flowering plants in spring appear red and blue-violet when blooming, while some broad-leaved forest species appear yellow in spring. The diversity of color patches extends the fixation and increases the frequency of the subjects. The addition of spring flowering plants and deciduous broad-leaved plants with yellow in spring, such as *Rhododendron* and *Populus davidiana*, can be effective in increasing the visual attractiveness of spring forest color. In summer, both evergreen and broad-leaved tree species are in the leaf-spreading stage, the forest color is predominantly green, and the characteristics color have limited impact on the visual indicators. In autumn, Grass proportion (GP) and Color patch density (PD) were positively correlated with AOI first fixation time (AFFT). That is to say, the smaller the proportion of grassland, the more sparsely distributed the color patches were, and the shorter the time for subjects to enter the autumn forest color for the first time. AOI total fixation duration (ATFD) and AOI fixation count (AFC) were closely related to the color characteristic indexes including yellow (H3, H4), green (H5, H6), and Shannon’s diversity index of color patch (SHDI). In autumn, some deciduous broad-leaved tree species showed yellow leaf organs and diversified forest colors due to phenological changes, thus extending the fixation time and increasing the frequency at which the subjects viewed autumn forest colors. The yellow plants have a significant impact on the visual attractiveness of autumn forest color. Thus, the reasonable addition of some deciduous broad-leaved tree species, such as *Acer forrestii* Diels, *Acer flabellatum* Rehd., *Acer oliverianum* Pax, can be effective in enhancing the visual attractiveness of autumn forest color. In winter, red (H1, H2), green (H5-9) and the number of colors (NC) make a huge difference to AOI first fixation time (AFFT). A few deciduous broad-leafed plants, such as *Castanea mollissima*, are red in winter. Differently, evergreen trees are green. Thus, bringing down the proportion of red and increasing the proportion of green can help shorten the time spent by the subjects first observing the area of interest. AOI total fixation duration (ATFD) and AOI fixation count (AFC) are closely associated with green (H6, H7), the number of Colors (NC) and Shannon’s diversity index of color patch (SHDI). In case of high color quantity, green proportion and patch diversity, forest color is more visually attractive. In this case, the proportion of evergreen species can be increased as appropriate, for example, *Quercus pannosa* Hand.-Mazz., *Cyclobalanopsis glaucoides* Schott. and other plants. Moreover, it is advisible to choose the plants of different color systems for overall planning, limit the concentration degree of plant planting, pay attention to diversified color patches, and create a more attractive color landscape.

### Rationality and restriction

#### Rationality and feasibility

This study is purposed to figure out the characteristics and differences in the evaluation of visual behavior and landscape preference when the participants make observation of forest color, based on which an exploration will be conducted into the factors of forest color characteristics that could exert influence on the visual behavior of participants through visual behavior experiment and landscape preference evaluation. This analysis is the focus of this study, which is aimed to introduce visual behavior experiment into forest color evaluation on the basis of prior studies on landscape cognition, thus providing basic research for the development of a more scientific, multi-dimensional and quantitative forest color evaluation system. In respect of research methods, eye movement analysis and SBE evaluation method are adopted for this study. It has been demonstrated in some studies that the application of eye tracking technology provides objective auxiliary support for the practice of landscape evaluation, with certain results achieved [48]. Having been validated in prior studies, the SBE method is considered to be universally reliable [4]. In addition, the studies carried out by some scholars through the combination of eye-movement tracking technology and other subjective evaluation methods have been verified [33, 49]. They provide a theoretical basis for the development and experimental design of this study. According to the experimental results, the research results are partially consistent with the prior studies. With the prior studies verified, we present a novel idea about the evaluation of forest color. In conclusion, it is believed as reasonable and feasible to study the combination of eye-tracking technology and landscape preference evaluation for evaluating forest color.

#### Restriction

The restrictions placed on experimental materials. One of the purposes of this study is to understand the color characteristics and differences of Jiaozi Mountain forest in different seasons, and to identify the color characteristics that can influence the visual behavior of the subjects. In the experimental materials, the forest color is presented in the form of static photos. Despite the capability of landscape photos to replace the scene landscape and its verification [50], illumination, terrain and other environmental factors will have impact on the presentation of color in the actual field survey, which can cause uncertainty in the attention and cognitive analysis of color by participants, thus affecting the effectiveness and reliability of the results. Virtual reality technology (VR), portable eye tracker and other emerging technologies have been commonly adopted by scholars. Virtual reality technology can be applied to present the outdoor landscape indoors to the best effect. Thus, the subjects can be organized to conduct the forest landscape visual tracking experiment. Providing technical support for field experiments, portable eye tracker can be more objective in reflecting the color perception of the subjects. In the future research, VR, portable eye tracker and other technologies will be applied to compare the effectiveness of optimized evaluation methods, thus expanding the breadth and depth of research on forest color evaluation.

The restrictions imposed on the extraction of color patches. In this study, the component elements of forest color were extracted by carrying out the self-designed program of the research group, with the color patches delineated by people. In practice, the accuracy of some colors in the course of human eye recognition is low due to the mix of colors. At present, as image recognition technology advances, it is now possible to define and describe the spatial composition of color using computer language. Therefore, in order for the further improvement of the forest color extraction method, multi-disciplines can be conducted to automatically identify the color patches under the same tolerance through the color threshold and computer language, thus achieving the automatic acquisition of forest color patches.

## Conclusions

Forest color evaluation is a combination of forest color characteristics and human cognitive. As the most vivid viewing element of forest landscape, color is very important to the improvement of forest visual quality. This study attempted to explore the relationship among color characteristics, landscape preference and visual behavior of forest color by combining eye tracking technology with subjective evaluation. Taking Jiaozi Mountain as the sample, the visual behavior and landscape preference of forest color landscape in different seasons were evaluated.

Our results are summarized as follows:

1. The color characteristics of Jiaozi Mountain in different seasons are obviously different, and the seasonal color changes are consistent with the plant species and seasonal characteristics. Spring and autumn are the most colorful, while summer and winter are relatively low in color richness.
2. From the perspective of landscape preference, subjects have different preferences for forest color landscape in different seasons. On the whole, the aesthetic quality of landscape is above the medium level, and the beauty of forest color landscape in winter is the lowest.
3. In terms of visual behavior evaluation, the visual focus and visual attention of the subjects vary with the color information contained in the forest landscape color. Visual behavior indexes are significantly correlated with many forest color characteristics indexes. Landscape areas with high color saturation, rich color number and high color patch heterogeneity are more likely to attract the attention of subjects.
4. In terms of visual behavior and landscape preference, the visual behavior of subjects in the annual color evaluation is highly correlated with landscape beauty. Annual images can help subjects identify scene information faster and generate cognitive preferences. In the single season forest color evaluation, there is no correlation between the first attention time and cognitive preference. Subjects will focus earlier on the most prominent color features. At the same time, the correlation between attention duration, attention frequency and cognitive preference only exists in spring, autumn and winter, and there is no correlation in summer when color difference is relatively small. Rich color information and visual attention are important factors affecting the correlation between visual behavior, cognitive preferences and forest color features. In different stages of landscape perception, the relationship between visual behavior and cognitive preferences is different. Landscape preference can interpret visual behavior index to some extent. However, excluding the above, they are still relatively independent indicators and cannot be replaced with each other. Landscape preference research is subjective and will be affected by the public’s personal aesthetic and experience, while visual behavior experiment is an objective physiological monitoring study with little subjective influence from the public, which is easy to monitor the changes in line of sight and obtain reliable behavioral data. Therefore, in future research, subjective preference and objective visual analysis can complement each other and provide a more comprehensive evaluation method for landscape evaluation.

In addition, in the practice of forest landscape management, the color modification and improvement of forest landscape is an effective way to increase the beauty of forest landscape. Comprehensive and reasonable control of the threshold range and combination of forest color characteristics can effectively improve the visual attractiveness of forest.
